# Validation List no. 217. Valid publication of new names and new combinations effectively published outside the IJSEM

**DOI:** 10.1099/ijsem.0.006275

**Published:** 2024-06-03

**Authors:** Aharon Oren, Markus Göker

**Affiliations:** 1 The Institute of Life Sciences, The Hebrew University of Jerusalem, The Edmond J. Safra Campus, 9190401 Jerusalem, Israel; 2 Leibniz Institute DSMZ – German Collection of Microorganisms and Cell Cultures, Inhoffenstrasse 7B, 38124 Braunschweig, Germany

The purpose of this list is to publish in the *International Journal of Systematic and Evolutionary Microbiology* (IJSEM) those new names and new combinations that were effectively published outside the IJSEM as set forth in Rule 27 of the International Code of Nomenclature of Prokaryotes (ICNP; 2022 Revision) [1]. Authors and other individuals wishing to have new names and/or combinations included in future lists should send an electronic copy of the published paper to the IJSEM Editorial Office for confirmation that all of the other requirements for valid publication have been met. It is also a requirement of IJSEM and the International Committee of Systematics of Prokaryotes that authors of new species, new subspecies and new combinations provide evidence that types are deposited in two recognized culture collections in two different countries. It should be noted that the date of valid publication of these new names and combinations is the date of publication of this list, not the date of the original publication of the names and combinations. The authors of the new names and combinations are as given below. Inclusion of a name on these lists validates the publication of the name and thereby makes it available in the nomenclature of prokaryotes. Inclusion of a name on these lists is part of the requirements of valid publication of an effectively published name. Indeed, some of these names may, in time, be shown to be synonyms, or the organisms may be transferred to another genus, thus necessitating the creation of a new combination.

**Table:** 

Name/authors	Proposed as	Nomenclatural type^1^	Priority^2^	Reference
*Acinetobacter suaedae* Xu *et al*. 2024, 4^3^	sp. nov.	C16S1 (=CGMCC 1.17074=KCTC 72456)	29	[2]
*Actinomyces acetigenes* Tian *et al*. 2023, 11^3^	sp. nov.	VPI D163E-3 (=ATCC 49340=CCUG 35339)^4^	23	[3]
*Actinomyces stomatis* Tian *et al*. 2023, 11^3^	sp. nov.	PK606 (=ATCC 51655=CCUG 33930)	23	[3]
*Alexandriicola* Zhu *et al*. 2022, 483	gen. nov.	*Alexandriicola marinus*	10	[4]
*Alexandriicola marinus* Zhu *et al*. 2022, 484	sp. nov.	LZ-14 (=CCTCC AB 2017230=KCTC 62342)	10	[4]
*Aquirufa regiilacus* Pitt *et al*. 2024, 8^3^	sp. nov.	LEOWEIH-7C (=DSM 116390=JCM 36347)	41	[5]
*Arenibacterium arenosum* Jeong *et al*. 2022, 5^3^	sp. nov.	CAU 1593 (=KCTC 82402=MCCC 1K05671)	8	[6]
*Bradyrhizobium prioriisuperbiae* corrig. Prescott *et al*. 2023, 1358^5^	sp. nov.	BL16A (=DSM 112479=LMG 33180=NCTC 14602)	6	[7]
*Bradyrhizobium roseum* corrig. Zhang *et al*. 2023, 10^3,6^	sp. nov.	S12-14-2 (=CGMCC 1.19422=JCM 34606)	30	[8]
*Brenneria uluponensis* corrig. Prescott *et al*. 2023, 1361^7^	sp. nov.	K61 (=DSM 116657=LMG 33184)	6	[7]
*Collinsella ureilytica* corrig. Oh *et al*. 2023, 8^3,8^	sp. nov.	AGMB00827 (=GDMCC 1.2724=KCTC 25287)	38	[9]
*Croceibacterium aestuarii* Sun *et al*. 2024, 12^3^	sp. nov.	D39 (=KCTC 82757=MCCC 1K07008)	27	[10]
*Deinococcus taeanensis* Lee *et al*. 2022, 6^3^	sp. nov.	TS293 (=JCM 34027=KCTC 43191)	31	[11]
*Denitromonas* Reyes-Umana and Coates 2023, 10^3^	gen. nov., nom. rev.	*Denitromonas iodatirespirans*	25	[12]
*Denitromonas halophila* Reyes-Umana and Coates 2023, 11^3^	sp. nov.	SFB-3 (=ATCC TSD-270=DSM 113305)	25	[12]
*Denitromonas iodatirespirans* Reyes-Umana and Coates 2023, 10^3^	sp. nov.	IR-12 (=ATCC TSD-242=DSM 113304)	25	[12]
*Denitromonas ohlonensis* Reyes-Umana and Coates 2023, 10^3,9^	sp. nov.	SFB-1 (=ATCC TSD-271=DSM 113310)	25	[12]
*Desulfoferula* Watanabe *et al*. 2023, 5^3^	gen. nov.	*Desulfoferula mesophila* corrig.	26	[13]
*Desulfoferula mesophila* corrig. Watanabe *et al*. 2023, 5^3,10^	sp. nov.	12FAK (=DSM 115219=JCM 39399)^11^	26	[13]
*Enterobacter pasteurii* Rahi *et al*. 2024, 11^3,12^	sp. nov.	A-8 (=ATCC 23355=DSM 26481)^13^	35	[14]
*Fusibacillus* Bai *et al*. 2023, 4^3^	gen. nov.	*Fusibacillus kribbianus*	18	[15]
*Fusibacillus kribbianus* Bai *et al*. 2023, 4^3^	sp. nov.	YH-rum2234 (=DSM 116041=KCTC 25710)^14^	18	[15]
*Hallella absiana* Bai *et al*. 2023, 6^3^	sp. nov.	YH-C38 (=JCM 35423=KCTC 25103)	19	[16]
*Haloarcula amylovorans* Ma *et al*. 2024, 8^3^	nom. nov. [basonym: *Halomicroarcula amylolytica* Chen *et al*. 2020]	LR21 (=CGMCC 1.13611=NBRC 113588)	28	[17]
*Haloarcula halobia* Ma *et al*. 2024, 11^3^	sp. nov.	XH51 (=CGMCC 1.17215=JCM 34149)	28	[17]
*Haloarcula halophila* Ma *et al*. 2024, 10^3^	sp. nov.	DFY41 (=CGMCC 1.18544=JCM 34479)	28	[17]
*Haloarcula laminariae* (Ma *et al*. 2023) Ma *et al*. 2024, 8^3^	comb. nov. [basonym: *Halomicroarcula laminariae* Ma *et al*. 2023]	LYG-108 (=CGMCC 1.13607=JCM 32950)	28	[17]
*Haloarcula limicola* (Zhang and Cui 2014) Ma *et al*. 2024, 8^3^	comb. nov. [basonym: *Halomicroarcula limicola* Zhang and Cui 2014]	YGHS32 (=CGMCC 1.12129=JCM 18640)	28	[17]
*Haloarcula litorea* Ma *et al*. 2024, 10^3^	sp. nov.	GDY20 (=CGMCC 1.18545=JCM 34480)	28	[17]
*Haloarcula marina* (Ma *et al*. 2023) Ma *et al*. 2024, 10^3^	comb. nov. [basonym: *Halomicroarcula marina* Ma *et al*. 2023]	DT1 (=CGMCC 1.18928=JCM 35414)	28	[17]
*Haloarcula nitratireducens* (Durán-Viseras *et al*. 2022) Ma *et al*. 2024, 10^3^	comb. nov. [basonym: *Halomicroarcula nitratireducens* Durán-Viseras *et al*. 2022]	F27 (=CECT 9636=JCM 33314)	28	[17]
*Haloarcula ordinaria* Ma *et al*. 2024, 12^3^	sp. nov.	ZS-22-S1 (=CGMCC 1.12565=JCM 30035)	28	[17]
*Haloarcula pelagica* Ma *et al*. 2024, 11^3^	sp. nov.	YJ-61-S (=CGMCC 1.12573=JCM 30034)	28	[17]
*Haloarcula pellucida* (Echigo *et al*. 2013) Ma *et al*. 2024, 10^3^	comb. nov. [basonym: *Halomicroarcula pellucida* Echigo *et al*. 2013]	BNERC31 (=CECT 7537=JCM 17820)	28	[17]
*Haloarcula rara* Ma *et al*. 2024, 11^3^	sp. nov.	SHR3 (=CGMCC 1.15231=JCM 30843)	28	[17]
*Haloarcula rubra* (Durán-Viseras *et al*. 2022) Ma *et al*. 2024, 10^3^	comb. nov. [basonym: *Halomicroarcula rubra* Durán-Viseras *et al*. 2022]	F13 (=CECT 9686=JCM 33313)	28	[17]
*Haloarcula salinisoli* (Durán-Viseras *et al*. 2022) Ma *et al*. 2024, 10^3^	comb. nov. [basonym: *Halomicroarcula salinisoli* Durán-Viseras *et al*. 2022]	F24A (=CECT 9687=CCM 8955)	28	[17]
*Halomicroarcula onubensis* Straková *et al*. 2024, 16^3^	sp. nov.	S3CR25-11 (=CCM 9254=CECT 30621)	42	[18]
*Halomicroarcula saliterrae* Straková *et al*. 2024, 15^3^	sp. nov.	S1CR25-12 (=CCM 9252=CECT 30620)	42	[18]
*Helicovermis* Miyazaki *et al*. 2024, 7^3^	gen. nov.	*Helicovermis profundi*	34	[19]
*Helicovermis profundi* Miyazaki *et al*. 2024, 7^3^	sp. nov.	S502 (=DSM 112048=JCM 39167)	34	[19]
*Herbidospora solisilvae* Yu *et al*. 2021, 588	sp. nov.	NEAU-GS84 (=CCTCC AA 2018041=JCM 33460)	1	[20]
*Infirmifilum* Zayulina *et al*. 2021, 12^3^	gen. nov.	*Infirmifilum lucidum*	32	[21]
*Infirmifilum lucidum* Zayulina *et al*. 2021, 12^3^	sp. nov.	3507LT (=KCTC 15797=VKM B-3376)	32	[21]
*Leucobacter edaphi* Zheng *et al*. 2023, 1442	sp. nov.	CSA2 (=CGMCC 1.18747=JCM 34360)	22	[22]
*Lichenifustis* Pankratov *et al*. 2023, 10^3^	gen. nov.	*Lichenifustis flavocetrariae*	4	[23]
*Lichenifustis flavocetrariae* Pankratov *et al*. 2023, 11^3^	sp. nov.	BP6-180914 (=KCTC 92872=UQM 41506=VKM B-3641)	4	[23]
*Lysobacter soyae* Kim *et al*. 2023, 15^3^	sp. nov.	CJ11 (=KACC 21716=NBRC 114478)	44	[24]
*Maribacter aquimaris* Zhang *et al*. 2023, 760^3^	sp. nov.	ANRC-HE7 (=KCTC 72532=MCCC 1K03787)	39	[25]
*Maribacter polysaccharolyticus* Gao *et al*. 2023, 12^3,15^	sp. nov.	D37 (=KCTC 82772=MCCC 1K06123)	20	[26]
*Mesorhizobium intechi* Estrella *et al*. 2020, 5^3^	sp. nov.	BD68 (=CECT 9304=LMG 30179)	7	[27]
*Microbacterium aurugineum* Lee *et al*. 2023, 16^3^	sp. nov.	KSW4-10 (=DSM 112583=KACC 22272)	5	[28]
*Microbacterium croceum* Lee *et al*. 2023, 16^3^	sp. nov.	SSW1-49 (=DSM 112581=KACC 22275)	5	[28]
*Microbacterium galbinum* Lee *et al*. 2023, 17^3^	sp. nov.	KSW2-24 (=DSM 112584=KACC 22276)	5	[28]
*Microbacterium sufflavum* Lee *et al*. 2023, 17^3^	sp. nov.	SSW1-47 (=DSM 112582=KACC 22279)	5	[28]
*Moraxella haemolytica* Li *et al*. 2024, 7^3^	sp. nov.	ZY171148 (=CCTCC AB 2021471=CCUG 75920)	24	[29]
*Mycobacterium salfingeri* Musser *et al*. 2022, 7^3,16^	sp. nov.	20–157661 (=DSM 113368=BCCM/ITM 501207)	33	[30]
*Nesterenkonia aerolata* Song *et al*. 2024, 7^3^	sp. nov.	LY-0111 (=GDMCC 1.3945=JCM 36375)	36	[31]
*Ochrobactrum chromiisoli* Yang *et al*. 2024, 11^3^	sp. nov.	YY2X (=CCTCC AB 2023035=JCM 36000)	21	[32]
*Ochrobactrum quorumnocens* Krzyźanowska *et al*. 2019, 13^3,17^	sp. nov.	A44 (=LMG 30544=PCM 2957)	3	[33]
*Paraflavitalea speifideaquila* corrig. Prescott *et al*. 2023, 1360^18^	sp. nov.	BL16E (=DSM 112478=LMG 33183=NCTC 14603)	6	[7]
*Paramicrobacterium* Lee *et al*. 2023, 15^3^	gen. nov.	*Paramicrobacterium agarici*	5	[28]
*Paramicrobacterium agarici* (Young *et al*. 2010) Lee *et al*. 2023, 16^3^	comb. nov. [basonym: *Microbacterium agarici* Young *et al*. 2010]	CC-SBCK-209 (=CCM 7686=DSM 21798)	5	[28]
*Paramicrobacterium chengjingii* (Zhou *et al*. 2021) Lee *et al*. 2023, 16^3^	comb. nov. [basonym: *Microbacterium chengjingii* Zhou *et al*. 2021]	HY60 (=CGMCC 1.17468=KACC 22102)^19^	5	[28]
*Paramicrobacterium fandaimingii* (Zhou *et al*. 2021) Lee *et al*. 2023, 16^3^	comb. nov. [basonym: *Microbacterium fandaimingii* Zhou *et al*. 2021]	HY82 (=CGMCC 1.17469=KACC 22101)^20^	5	[28]
*Paramicrobacterium humi* (Young *et al*. 2010) Lee *et al*. 2023, 16^3^	comb. nov. [basonym: *Microbacterium humi* Young *et al*. 2010]	CC-12309 (=CCM 7687=DSM 21799)	5	[28]
*Pararhizobium gei* (Shi *et al*. 2016) Zhang *et al*. 2024, 7^3^	comb. nov. [basonym: *Rhizobium gei* Shi *et al*. 2016]	ZFJT-2 (=CCTCC AB 2013015=LMG 27603)^21^	17	[34]
*Pararhizobium qamdonense* Zhang *et al*. 2024, 6^3^	sp. nov.	T808 (=CICC 25216=JCM 36247)	17	[34]
*Pelagibacterium flavum* Chen *et al*. 2024, 5^3^	sp. nov.	YIM 151497 (=CGMCC 1.61521=KCTC 49826=MCCC 1K08053)	37	[35]
*Phycicoccus sonneratiae* corrig. Tang *et al*. 2023, 6^3,22^	sp. nov.	MQZ13P-5 (=CGMCC 1.18744=JCM 34337)^23^	15	[36]
*Rhodobacter kunshanensis* Liu *et al*. 2021, 3795	sp. nov.	HX-7–19 (=CCTCC AB 2020148=KCTC 72471)	13	[37]
*Rhodobacter xinxiangensis* Han *et al*. 2020, 1746	sp. nov.	TJ48 (=CCTCC AB 2019120=KCTC 72510)	11	[38]
*Saccharopolyspora oryzae* Kammanee *et al*. 2023, 663	sp. nov.	WRP15-2 (=NBRC 115560=TBRC 15728)	2	[39]
*Sedimentimonas* Mu *et al*. 2022, 991	gen. nov.	*Sedimentimonas flavescens*	40	[40]
*Sedimentimonas flavescens* Mu *et al*. 2022, 991	sp. nov.	B57 (=CGMCC 1.19488=KCTC 92053)	40	[40]
*Selenobaculum* Yeo *et al*. 2023, 10^3,24^	gen. nov.	*Selenobaculum gibii* corrig.	16	[41]
*Selenobaculum gibii* corrig. Yeo *et al*. 2023, 10^3,25^	sp. nov.	ICN-92133 (=JCM 36070=KCTC 25622)	16	[41]
*Sphingomonas donggukensis* Kim *et al*. 2023, 6^3^	sp. nov.	RMG20 (=KACC 22358=TBRC 15162)	43	[42]
*Sphingomonas liriopis* Kim *et al*. 2023, 6^3^	sp. nov.	RP10 (=KACC 22357=TBRC 15161)	43	[42]
*Sphingomonas tagetis* Kim *et al*. 2023, 7^3^	sp. nov.	MG17 (=KACC 22355=TBRC 15160)	43	[42]
*Thermohalobaculum* Pan *et al*. 2021, 1826	gen. nov.	*Thermohalobaculum xanthum*	12	[43]
*Thermohalobaculum xanthum* Pan *et al*. 2021, 1826	sp. nov.	M0105 (=KCTC 52118=MCCC 1K03767)^26^	12	[43]
*Xinfangfangia pollutisoli* Mu *et al*. 2022, 4^3^	sp. nov.	Clo-40 (=GDMCC 1.2845=KCTC 92089)^27^	9	[44]
*Zhongshania ponticola* Park *et al*. 2018, 1181^28^	sp. nov.	GM-8 (=NBRC 113193=KACC 19616=KCTC 62425)	14	[45]

For references to Validation Lists 1–71, see *Int J Syst Bacteriol* **49** (1999) 1325. Lists 72–197 were published in *Int J Syst Evol Microbiol* **50** (2000) 3, 423, 949, 1415, 1699, 1953; and **51** (2001) 1, 263, 793, 1229, 1619, 1945; and **52** (2002) 3, 685, 1075, 1437, 1915; and **53** (2003) 1, 373, 627, 935, 1219, 1701; and **54** (2004) 1, 307, 631, 1005, 1425, 1909; and **55** (2005) 1, 547, 983, 1395, 1743, 2235; and **56** (2006) 1, 499, 925, 1459, 2025, 2507; and **57** (2007) 1, 433, 893, 1371, 1933, 2449; and **58** (2008) 1, 529, 1057, 1511, 1993, 2471; and **59** (2009) 1, 451, 923, 1555, 2129, 2647; and **60** (2010) 1, 469, 1009, 1477, 1985, 2509; **61** (2011) 1, 475,1011, 1499, 2025, 2563; and **62** (2012) 1, 473, 1017, 1443, 2045, 2549; and **63** (2013) 1, 797, 1577, 2365, 3131, 3931; and **64** (2014) 1, 693, 1455, 2184, 3603; and **65** (2015), 1, 741, 1112, 2017, 2777, 3767; and **66** (2016) 1, 1603, 1913, 2463, 3761, 4299; and **67** (2017) 1, 529, 1095, 2075, 3140, 4291; and **68** (2018), 1, 693, 1411, 2130, 2707, 3379; and **69** (2019), 5, 597, 1247, 1844, 2627, 3313, and **70** (2020) 1, 1443, 2960, 4043, 4844, 5596. Lists 197–216 were published in *Int J Syst Evol Microbiol* **71** (2021) 004600, 004688, 004773, 004846, 004943, 005096; and **72** (2022) 005167, 005260, 005331, 005422, 005517, 005592; and **73** (2023) 005709, 005812, 005845, 005931, 005997, 006080; and **74** (2024) 006173, 006229.

1Abbreviations of culture collections cited in this list can be found at https://lpsn.dsmz.de/text/collections

2Priority number assigned according to the date the documentation and request for validation are received.

3The journal in which the name was effective published does not have continuous pages.

4The effective publication states that the type strain was also deposited as CCUG 34286, but no documentation was supplied.

5The list editors have corrected the epithet to *prioriisuperbiae* (pri.o.ri.i.su.per’bi.ae. N.L. fem. n. *prioria*, a priory; L. fem. n. *superbia*, pride; N.L. gen. fem. n. *prioriisuperbiae*, of Priory Pride …).

6The list editors have corrected the epithet to *roseum* (ro’se.um. L. neut. adj. *roseum*, rose-colored, pink).

7The list editors have corrected the epithet to *uluponensis* (u.lu.po.nen’sis. N.L. fem. adj. *uluponensis*, originating from Ulupō, …).

8The list editors have corrected the epithet to *ureilytica* (u.re.i.ly’ti.ca. N.L. fem. n. *urea*, urea; N.L. masc. adj. *lyticus* (from Gr. masc. adj. *lytikos*), able to loosen, able to dissolve; N.L. fem. adj. *ureilytica*, urea-dissolving).

9The following syllabification and etymology are preferred: oh.lo.nen’sis. N.L. fem. adj. *ohlonensis*, belonging to the Ohlone land.

10The list editors have corrected the epithet to *mesophila* (me.so’phi.la. N.L. fem. adj. *mesophila*, mesophilic).

11The protologue states DSMZ 115219 instead of DSM 115219.

12The protologue heading should state *Enterobacter* instead of *E.*

13The effective publication states that the type strain was also deposited as CIP 103550, CCUG 33777, CECT 5075, WDCM 00082 and NCTC 13380, but no documentation was supplied.

14The protologue states DSMZ 116041 instead of DSM 116041.

15The protologue heading should state sp. nov. instead of sp. Nov., and following etymology is preferred: *…* Gr. masc. adj. *polys*, many; Gr. neut. n. *sakcharon*, sugar; Gr. masc. n. *lytikos*, dissolving; N.L. masc. adj. *polysaccharolyticus*, …).

16The following syllabification and etymology are preferred: sal.fin’ge.ri. N.L. gen. n. *salfingeri*…, for the pioneering contributions of Dr. Max Salfinger,

17The epithet looks like Latin but is malformed. *Quorum* is a derived form of the pronoun *qui*, in the genitive plural, meaning ‘whose’, ‘of whom’, and *nocere* does not mean ‘quench’ but ‘hurt’. Moreover, the guidelines of Appendix 9 of the ICNP for the formation of compound epithets were not followed. The epithet *multitudinisentens* was earlier used to mean ‘quorum sensing’.

18The list editors have corrected the epithet to *speifideaquila* (spe.i.fi.de.a’qui.la. L. fem. n. *spes*, hope; L. fem. n. *fides*, faith; L. fem. n. *aquila*, eagle; N.L. fem. n. *speifideaquila*, the eagle of hope and faith).

19The effective publication states that the type strain was also deposited as GDMCC 1.1951, but no documentation was supplied.

20The effective publication states that the type strain was also deposited as GDMCC 1.1949, but no documentation was supplied.

21The effective publication states that the type strain was also deposited as KCTC 32301, but no documentation was supplied.

22The list editors have corrected the epithet to *sonneratiae* (son.ne.ra’ti.ae. N.L. gen. n. *sonneratiae*, of *Sonneratia* …).

23The JCM accession number of the type strain is 34337 and not 34,337 as given in the protologue.

24The following syllabification and etymology are preferred: Se.le.no.ba’cu.lum. Gr. fem. n. *selene*, the moon; L. neut. n. *baculum*, a stick, a rod; N.L. neut. n. *Selenobaculum*, a rod-shaped bacterium belonging to the family *Selenomonadaceae*.

25The list editors have corrected the epithet to *gibii* (gi’bi.i. N.L. gen. n. *gibii*, of GBI, acronym for Green-Bio Institute, where the type strain was isolated).

26The effective publication states that the type strain was also deposited as BGMRC 2019 and NBRC 112057, but no documentation was supplied.

27The KCTC accession number of the type strain is 92089 and not 92,089 as given in the protologue.

28The protologue heading should state *Zhongshania ponticola* instead of *Z. ponticola*.

The following correction should be made in Validation List 215 [46]: The type strain of *Luteibacter aegosomaticola* Joe *et al*. 2023 was deposited as JCM 35472 and KCTC 92393, and not as JCM 35471 and KCTC 92392.
